# Inflammation, But Not Telomere Length, Predicts Successful Ageing at Extreme Old Age: A Longitudinal Study of Semi-supercentenarians

**DOI:** 10.1016/j.ebiom.2015.07.029

**Published:** 2015-07-29

**Authors:** Yasumichi Arai, Carmen M. Martin-Ruiz, Michiyo Takayama, Yukiko Abe, Toru Takebayashi, Shigeo Koyasu, Makoto Suematsu, Nobuyoshi Hirose, Thomas von Zglinicki

**Affiliations:** a Centre for Supercentenarian Research, Keio University School of Medicine, Tokyo, Japan; b Newcastle University Institute for Ageing, Campus for Ageing and Vitality, Newcastle University, UK; c Centre for Preventive Medicine, Keio University School of Medicine, Tokyo, Japan; d Department of Preventive Medicine and Public Health, Keio University School of Medicine, Tokyo, Japan; e Department of Biochemistry, Keio University School of Medicine, and JST, ERATO, Suematsu Gas Biology Project, Tokyo, Japan; f Laboratory for Immune Cell Systems, RIKEN Centre for Integrative Medical Sciences (IMS), Yokohama, Kanagawa, Japan; g Department of Microbiology and Immunology, Keio University School of Medicine, Tokyo, Japan

**Keywords:** IL-6, interleukin 6, TNF-alpha, tumour necrosis factor-alpha (TNF-alpha), CMV, cytomegalovirus, LTL, leukocyte telomere length, TOOTH, Tokyo Oldest Old Survey on Total Health, TCS, Tokyo Centenarians Study, JSS, Japanese Semi-Supercentenarians Study, CRP, C-reactive protein, NK cells, natural killer cells, CD, cluster of differentiation, AST, aspartate aminotransferase or aspartate transaminase, ALT, alanine aminotransferase or alanine transaminase, GGTP, gamma-glutamyl-transpeptidase, IQR, inter-quartile range, SD, standard deviation, ANOVA, analysis of variance, ELISA, enzyme-linked immunosorbent assay, PCR, polymerase chain reaction, MMSE, Mini-Mental State Examination, CVD, cardiovascular disease, eGFR, estimated glomerular filtration rate, Centenarian, Ageing, Inflammation, Telomere

## Abstract

To determine the most important drivers of successful ageing at extreme old age, we combined community-based prospective cohorts: Tokyo Oldest Old Survey on Total Health (TOOTH), Tokyo Centenarians Study (TCS) and Japanese Semi-Supercentenarians Study (JSS) comprising 1554 individuals including 684 centenarians and (semi-)supercentenarians, 167 pairs of centenarian offspring and spouses, and 536 community-living very old (85 to 99 years). We combined z scores from multiple biomarkers to describe haematopoiesis, inflammation, lipid and glucose metabolism, liver function, renal function, and cellular senescence domains. In Cox proportional hazard models, inflammation predicted all-cause mortality with hazard ratios (95% CI) 1.89 (1.21 to 2.95) and 1.36 (1.05 to 1.78) in the very old and (semi-)supercentenarians, respectively. In linear forward stepwise models, inflammation predicted capability (10.8% variance explained) and cognition (8^.^6% variance explained) in (semi-)supercentenarians better than chronologic age or gender. The inflammation score was also lower in centenarian offspring compared to age-matched controls with Δ (95% CI) = − 0.795 (− 1.436 to − 0.154). Centenarians and their offspring were able to maintain long telomeres, but telomere length was not a predictor of successful ageing in centenarians and semi-supercentenarians. We conclude that inflammation is an important malleable driver of ageing up to extreme old age in humans.

## Highlights

• We identify potential drivers of multiple dimensions of the ageing process up to 110 years of age.
• Inflammation is the prime candidate amongst potential determinants of mortality, capability and cognition up to extreme old age.
• Telomere length does not predict successful ageing in the very and extreme old, but centenarians and their offspring maintain telomeres better than non-centenarians.

Centenarians are less susceptible to the diseases, functional losses and dependencies of old age than the general public. Why centenarians age differently, i.e. which biological and pathophysiological processes drive ageing at this extreme old age, is not known. We found that chronic systemic inflammation becomes increasingly associated with risk of death, loss of cognitive function and increasing dependency, but did not predict multimorbidity. In contrast, long telomeres might be important to become a centenarian but did not predict the ageing process further on. Suppression of chronic inflammation could be an essential step towards further improvements in human healthy lifespan.

## 1 Introduction

With continuously increasing life expectancy, the impact of chronic diseases and dependencies, for which no specific cures are available, also increases. This leads to an enormous burden to individuals and societies alike, which threatens to overwhelm conventional curative and care models in medicine. Unfortunately healthy life expectancy, the number of years spent free of disabilities, has grown slower than life expectancy in a number of countries. Within the European Union, for instance, life expectancy at age 50 has grown by 1.2 years between 2005 and 2010, but healthy life expectancy has only grown slightly (0.5 years in men, 0.4 years in women) in the same time frame (Fouweather et al., 2015). Moreover, this has been accompanied by increasing inequalities between EU countries in healthy life span, driven preferentially by material deprivation and long-term unemployment rates (Fouweather et al., 2015).

Centenarians and (semi-)supercentenarians are regarded as model cases for ‘successful ageing’. Centenarians retain independence and capability as well as cognition at higher levels for longer than the general population, together with postponed mortality (Andersen et al., 2012, Arai et al., 2014, Pavlidis et al., 2012). Understanding the factors determining extreme longevity and compression of morbidity might help to achieve extended healthy life span for the wider population and to close the gap between the fastest and the slowest ageing population groups. Moreover, the proportion of centenarians and (semi-)supercentenarians in the population is growing fast making understanding of the relative importance of the factors that determine successful ageing at extreme age a relevant objective in its own right.

Such understanding cannot simply be extrapolated from results obtained in younger populations because the power of biomarkers to predict the impact of various pathophysiological mechanisms on successful ageing is frequently dependent on age itself (Martin-Ruiz and von Zglinicki, 2014). For late nonagenarians and centenarians, some relatively small studies have shown improved or at least well maintained levels of certain biomarkers including immunosenescence markers (Strindhall et al., 2007) but data on telomere length, a prominent marker of cell senescence, were contradictory in this age group (Atzmon et al., 2010, Kimura et al., 2007, Tedone et al., 2014). In contrast, chronic inflammation is a strong negative predictor of successful ageing in younger cohorts (Jenny et al., 2012) and a cause of accelerated ageing in mice (Jurk et al., 2014) but appears to lose predictive power in centenarians (Gangemi et al., 2005). On this basis, it has been suggested that inflammation might change its role towards a pro-longevity factor at extreme old age (Salvioli et al., 2013).

Little is known about changes in biomarkers as centenarians progress towards even higher age. To our knowledge, there is only one small study reporting changes in telomere length between Italian centenarians and semi-supercentenarians (Tedone et al., 2014), which included only 29 semi-supercentenarians. So far, nothing is known about the relative power of biomarkers delineating different pathophysiological domains to predict successful ageing in centenarians and (semi-)supercentenarians.

Through a nation-wide recruitment effort, we established unique cohorts of centenarians and (semi-)supercentenarians with a maximum of 10-year mortality follow-up to identify genetic and biological pathways to healthy ageing and longevity using both cross-sectional and longitudinal approaches (Arai et al., 2014). These unique cohorts are supported by cohorts representing the oldest non-centenarians (Arai et al., 2010) as well as centenarian offspring and their spouses. We obtained quantitative measures of successful ageing, namely capability (Barthel index), multi-morbidity (disease count), cognitive function (MMSE score), and mortality (survival time). In addition, we generated biomarker data covering multiple relevant domains, which were each characteristic of a pathophysiological process with the potential to drive ageing. These domains are haematopoiesis/anaemia, inflammation, lipid and glucose metabolism, liver function, renal function, and cell-/immuno-senescence. This allowed us to address two important questions: i) which biological processes (measured by biomarker domain) are the most important predictors of successful ageing at extreme age? and ii) are the same processes active in both centenarians/ (semi-)supercentenarians and their offspring?

Together, our results suggest that suppression of inflammation is the most important driver of successful longevity that increases in importance with advancing age and is amenable to pharmacological intervention.

## 2 Methods

### 2.1 Study Population

The analytical cohort of this study comprised 1554 individuals including: 684 centenarians (100–104 years old) and (semi-)supercentenarians (105 years or older), 167 pairs of offspring and unrelated family (spouse of offspring) of centenarians, and 536 community-living very old (85 to 99 years), covering a wide range of chronological ages from around 50 up to 115 years. Sample size was primarily determined by availability.

Written informed consent to participate was obtained either from the participants or proxy when individuals lacked the capacity to consent. The study was approved by the Ethical Committee of the Keio University School of Medicine.

Samples of centenarians were derived from two prospective cohorts, the Tokyo Centenarians Study (TCS) and the Japanese Semi-supercentenarians Study (JSS). Details of cohort recruitment were described previously (Arai et al., 2010, Gondo et al., 2006, Takayama et al., 2007). The TCS took place from July 2000 to May 2002, and 304 centenarians living in the Tokyo metropolitan area (65 men, 239 women) were recruited based on the basic resident registry. Amongst them, 255 centenarians (58 men, 197 women, median age 100.7, IQR; 100.2–102.1 years) with enough DNA sample for telomere measurements were included in this analysis. The JSS is a nationwide longitudinal survey that involves mainly (semi-)supercentenarians, individuals aged 105 years or older. Between September 2002 and November 2011, a total of 429 centenarians (51 men, 378 women, median age 106.7 years, IQR; 105.6–107.6 years, 90.2% were older than 105 years) were enrolled in the JSS. As the main aim of JSS is to identify longevity-assurance genes, DNA samples to perform telomere length measurements were available from all JSS participants. On the basis of the Japanese cohort life tables, 122 and 617 men and women, respectively, per 100,000 births reached 100 years of age in 2000, while there were 12 and 90 per 100,000 births to reach an age of 105 in 2005, and the ratios to reach 110 years in 2010 were 0.3 and 6.6 per 100,000 births. Indeed only 13.9% and 0.9% of those who reached 100 years old then survived to 105 and 110 years old, respectively. Therefore, we examined associations between biomarkers and outcome indicators separately according to age at recruitment. During the recruitment process, we also recruited a first-degree offspring of centenarians (n = 167) and their spouses (n = 167) as unrelated family. In the Japanese tradition, elder sons are the most likely carers of their parents, thus 75.4% of offspring were male and exactly the same rate were female in the unrelated family cohort. Samples of the very old were derived from a community-based prospective cohort of the Tokyo Oldest Old Survey on Total Health (TOOTH). The design, recruitment, and entire procedure of the TOOTH were previously described (Arai et al., 2014). Briefly, between March 2008 and November 2009, we recruited a randomly selected sample of 984 inhabitants of Tokyo aged 85 years or older for face-to-face interviews at the participants' residences, of which 542 (236 men, 306 women; mean ± SD age, 87.8 ± 2.2 years; range, 85–102 years) participated in extended medical examination. Amongst them, 2 individuals aged 100 years or older and 4 lacking a DNA sample were excluded, leaving 536 to be enrolled in this analysis.

Participants were assessed and examined by trained geriatricians (N.H., Y.A., and M.T.) according to protocols described (Arai et al., 2014, Arai et al., 2010, Gondo et al., 2006, Takayama et al., 2007).

### 2.2 Outcomes

The outcomes of interest were cognitive status, capability, multi-morbidity and death. In the extreme old, these scalable variables are more informative than dichotic variables (e.g., frailty, diagnosed dementia). We established death from any cause (all centenarians and TOOTH participants) and death from cardiovascular disease (CVD), cancer, and pneumonia (TOOTH participants only). For both the TCS and JSS cohorts, information on mortality was ascertained by telephone contact or mail survey conducted every 12 months until 30 September 2012 with a 249,548 person-day (median period, 752 days; range, 3–4143 days) follow-up for the TCS and a 248,846 person-day (median period, 503 days; range, 1–2561 days) follow-up for the JSS. During follow-up, there were 249 (97.6%) deaths, one participant (0.4%) was alive, and 5 (2.0%) were lost to follow-up in the TCS cohort. In JSS there were 375 (87.4%) deaths, 45 participants (10.5%) were alive, and 9 (2.1%) were lost to follow-up. Most of the (semi-)supercentenarians who remained in the cohort were those who were enrolled in recent years. For the TOOTH cohort (85–99 years at enrollment), participants were followed up for all-cause and cause-specific mortality by telephone contact or mail survey conducted every 12 months (Iinuma et al., 2015). At 3 years, those who remained in the cohort were examined according to the same protocol as in the baseline survey (first follow-up survey). Thereafter, the mortality survey was extended and 4 year-follow-up was completed by the end of November 2013. Total follow-up time was 669,390 person-days (median period, 1460 days; range, 27–1460 days). There were 123 (22.9%) deaths, amongst them 32 related to cancers, 36 to CVD, 25 to pneumonia, 17 to advanced age, 4 to severe dementia, and 9 by unknown causes.

Additional outcomes of interest were cognitive function which was evaluated according to the Mini-Mental State Examination (MMSE), capability (assessed using the Barthel Index) and multi-morbidity. Information regarding past medical history was obtained from participants or care giver/proxy when participants lacked capacity, and from available documentations including discharge summary and medication list. The classification of medical conditions was based on the International Classification of Diseases, 10th Revision. A total of 8 chronic conditions, i.e. hypertension [I10], coronary heart disease [I20–I25], stroke [I60–I69], Diabetes mellitus [E11], hyperlipidaemia [E78], fragility fracture [M80], cancer (excluding skin cancer, [C00–C41, C45–67]) and renal disease [N00–N19], were incorporated into the multimorbidity count. Chronic kidney disease was not included because its diagnosis in the oldest old has not been validated.

### 2.3 Biomarkers

Non-fasting blood samples were obtained at baseline and were stored at − 80 °C until subsequent assay. Total DNA was extracted from whole blood by using the FlexGene DNA Kit (QIAGEN, Hilden, Germany) and was stored in FG3 solution at 4 °C (Kojima et al., 2004). Candidate biomarkers were measured as follows:

Telomere length: A total of 1554 samples from the 3 cohorts included in this study were assessed, all by the same lab. DNA concentration and quality were monitored by agarose gel electrophoresis. No sample had to be discarded because of DNA degradation. Telomere length was measured as abundance of telomeric template versus a single copy gene (36B4) using the following primers: TelA (5′-CGG TTT GTT TGG GTT TGG GTT TGG GTT TGG GTT TGG GTT-3′); TelB (5′-GGC TTG CCT TAC CCT TAC CCT TAC CCT TAC CCT TAC CCT-3′); 36B4F (5′-CAG CAA GTG GGA AGG TGT AAT CC 3′) and 36B4R (5′-CCC ATT CTA TCA TCA ACG GGT ACA A-3′). Three internal control DNA samples of know telomere length (10.4 kb, 3.9 kb and 2 kb) were run within each plate to correct for plate–to-plate variation. Measurements were performed in triplicate. All PCRs were carried out on an Applied Biosystems 7900HT Fast Real Time PCR system with 384-well plate capacity. The intra-assay coefficient of variation was 2.7% while the inter-assay coefficient of variation was 5.1%. As a further authentication of our telomere length measurements, we performed 2 revaluation exercises: because of the extreme longevity of our participants and the likelihood and relevance of short telomere length values, samples that were in either the 5% top or 5% bottom of the telomere lengths distribution as well as those samples that gave no valid data on the first run were re-examined. Throughout, first assessments of telomere length were confirmed and the final set of telomere measurements included valid data for 1541 participants (excluding 13 participants for which no valid data could be generated in two independent experiments). Furthermore, the impact of batch-to-batch (plate-to-plate) variation was examined by recombining a set of 192 DNA samples from centenarians and (semi-)supercentenarians in an independent order onto fresh 96-well plates and re-measuring them. This validated the absence of significant plate-to-plate bias and specifically confirmed longer telomeres in centenarians and (semi-)supercentenarians than expected from the regression measured at younger ages.

Further biomarkers: Cytomegalovirus (CMV) immunoglobulin G (IgG) antibody titers were measured by an enzyme immunoassay kit (Denka Seiken Co, Ltd., Tokyo Japan) using Behring ELISA processor III (Siemens Health Care Diagnostics, Tokyo, Japan). A sample was categorized as seropositive at a relative signal intensity of 2.0 or greater. Inter-assay CV of CMV IgG titer was 5.66%. Plasma levels of interleukin-6 (IL-6), and tumour necrosis factor-alpha (TNF-alpha) were measured in duplicate using commercially available ELISA kits [Quantikine HS (Human IL-6), R&D Systems, Minneapolis, U.S.A; Quantikine HS (Human TNF-alpha), R&D Systems, Minneapolis, U.S.A; respectively]. Inter-assay CVs of IL-6 and TNF-alpha were 9.43%, and 8.72%, respectively. Concentrations of C-reactive protein (CRP) were measured using a standard assay procedure in conjunction with a completely automated system (SRL Limited, Tokyo, Japan).

Serum creatinine-based eGFR was calculated as below according to Clinical Practice Guidebook for Diagnosis and Treatment of Chronic Kidney Disease 2012 (Japanese Society of Nephrology):

$$\mathrm{eGFR}\;\left(\mathrm{ml}/\min/1.73m^{2}\right)=194\times Cre{\left(\mathrm{mg}/\mathrm{dl}\right)}^{-1.094}\times {\mathrm{Age}}^{-0.287}\left(\times 0\;.739\;\mathrm{if}\;\mathrm{female}\right)\text{.}$$

To analyse lymphocyte subset markers, whole blood was collected in tubes containing heparin from 17 pairs of offspring and unrelated family, 8 centenarians and 141 (semi-) supercentenarians, including 27 participants who were enrolled for TCS at an age of 100–104 years but had the blood sample for lymphocyte phenotyping taken only after they reached 105 years. Accordingly, these 27 participants were included in the (semi-)supercentenarian group for analysis of lymphocyte markers (including the immunosenescence index), but were analysed as members of the centenarian group with respect to all other markers. Direct immunofluorescence of lysed whole blood was performed using flow cytometry (BD FACSCalibur™, BD Bioscience, New Jersey, U.S.A.) after labelling with fluorochrome conjugated monoclonal antibodies: anti-CD4-FITC, anti-CD8-PE, anti-CD16-FITC, anti-CD28-FITC, and CD56-PE (Beckman Coulter, Tokyo, Japan). Twenty thousand light-scatter (FSC versus SSC) gated events were analysed, and results were expressed as percentages of total lymphocytes.

### 2.4 Domain Score Calculation

Biomarkers that showed a strongly skewed distribution (i.e. CMV, IL-6, TNF-alpha, CRP, aspartate aminotransferase (AST), alanine aminotransferase (ALT) and gamma-glutamyl-transpeptidase (GGTP)) were log- converted (natural logarithm).

Data on biomarkers were further organized into domain scores by linearly combining Z-scores for individual biomarkers as follows:

1. Haematopoiesis: red blood cell count + haematocrit + white blood cell count.
2. Inflammation: CMV titer + IL-6 + TNF-alpha + CRP.
3. Lipid and glucose metabolism: low-density level (LDL) cholesterol + total cholesterol + glycated haemoglobin (HbA1c).
4. Liver function: AST + ALT + GGTP.
5. Kidney function: estimated glomerular filtration rate (eGFR).
6. Cell senescence: LTL (inverted).
7. Immunosenescence (105 + group only): LTL (inverted) + CD4/CD8 (inverted) + CD16 + CD28 (inverted) + CD56 for cases with available data.

CMV infection may contribute both to chronic inflammation and to cell senescence (Pawelec et al., 2009). Therefore, alternatively the inflammation score was calculated as IL-6 + TNF-alpha + CRP and the cell senescence score as CMV–LTL. Similarly, there are different opinions as to whether the monocyte marker CD16 and the NK cell marker CD56 may be regarded as immunosenescence markers (Hayhoe et al., 2010) and thus the immunosenescence score was alternatively calculated omitting these two markers. None of the conclusions in this paper was sensitive to these alternatives (data not shown).

### 2.5 Statistical Analysis

Baseline characteristics are expressed as means and standard deviations (SD) or as percentages. Continuous variables with a skewed distribution are described as medians [interquartile ranges (IQR)], and log-transformed for statistical analyses. Differences between baseline characteristics of participants within each age category except centenarians' offspring were analysed using the Cochrane–Armitage test for trend for proportions and analysis of variance for continuous measures.

For longitudinal analysis of age-specific survival probability, we plotted Kaplan-Meier mortality curves according to tertiles of each individual biomarker or each biomarker domain. Because telomere length, inflammatory biomarkers (i.e. interleukin-6, TNF-alpha) and CMV IgG titers differed substantially among the very old (85–99 years), centenarians (100–104 years), and (semi)-supercentenarians (105 + years), analyses were performed separately in these groups using age group-specific cut-offs of tertiles. A prognostically significant result was defined as log-rank *p* < 0.05. We used the Cox proportional hazards model to assess the multivariate-adjusted relative risk for the biomarker domains. In the multivariate analysis, factors known to be associated with mortality of the very old were included (age, gender, education, history of cardiovascular disease, hypertension, hyperlipidaemia, diabetes mellitus, and serum albumin level). Because female participants are dominant in centenarians, and distribution of smokers, especially current smokers, were remarkably skewed towards men, we did not include smoking status in our multiple models. Missing values were excluded from the respective analyses. For 27 participants of the centenarian group lymphocyte phenotyping was only performed after they had turned 105 years. For lymphocyte markers and immunosenescence analyses only, these participants are therefore included in the 105 + group, and were dichotomized by medians because of small numbers of individuals indicated.

We performed automated linear modelling with forward stepwise model selection using as target variable each of the outcome indicators Barthel Index, MMSE or multimorbidity (as number of chronic conditions). Age, gender and all domain scores available in the age group were included as predictors. Analyses were performed in each age group separately and over all unrelated age groups combined (excluding centenarian offspring).

Differences in domain scores between centenarian offspring and their spouses were assessed by ANOVA for all groups followed by Tukey post-hoc tests.

All analyses were performed using SPSS ver. 19.0 (SPSS, Chicago, IL, USA). Results were considered statistically significant at a *p* value of < 0.05 and two-sided tests were applied.

## 3 Results

### 3.1 Baseline Characteristics of the Cohorts

Table 1 shows the baseline characteristics of the participants stratified by age at recruitment. We found a general increase with age in CMV titer and inflammatory biomarkers and there was a loss of capability and cognition in centenarians and (semi-)supercentenarians. In contrast, cardiometabolic risk factors including hypertension, diabetes, and hyperlipidaemia actually improved at extreme age.

**Fig. 1:**
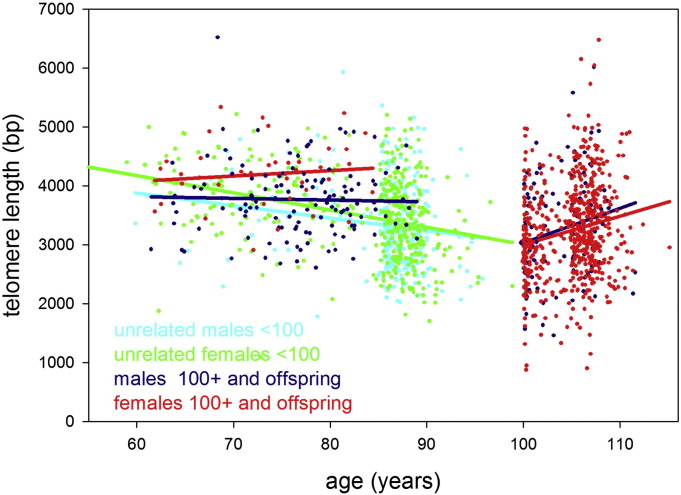
Telomere length in study participants up to 115 years of age. Leukocyte telomere length vs age is shown for males (blue or cyan) and females (green or red). Centenarians, (semi-)supercentenarians, and centenarian offspring are shown in blue (males) or red (females), respectively. Unrelated participants younger than 100 years are indicated in cyan (males) or green (females). Regression lines belonging to these groups are indicated by the same colour.

Despite low numbers, supercentenarians tended to display more ‘youthful’ values for many of the biomarkers tested. This trend reversal occurred even earlier (in the semi-supercentenarians) for two immunosenescence markers, i.e. the CD4/CD8 ratio, confirming findings in a small group of Swedish centenarians (Strindhall et al., 2007), and telomere length (Table 1). In unrelated individuals, telomeres shortened with age up to 100 years at rates of 21 ± 8 (males) and 29 ± 4 (females) bp/year (Fig. 1). However, after 100 years of age, telomeres increased in length by 59 ± 25 (males) and 48 ± 11 (females) bp/year. Interestingly, telomeres in centenarian offspring were maintained for more than 20 years at a length corresponding to 60 years of age in the general population (Fig. 1). In consequence, telomeres from centenarians, their offspring, and, especially, from (semi-)supercentenarians were significantly longer than expected for their age (Supplementary Figure S1). Paternal interfamilial correlations for telomere length between centenarians and their offspring were significant (r = 0.401; *p* = 0.031), but not maternal ones (Supplementary Figure S2).

### 3.2 Associations Between Biomarker Domains and Successful Ageing

We regarded capability (Barthel index), cognitive function (MMSE score), multimorbidity (disease count), and mortality (survival time) as informative descriptors of functional healthspan or successful ageing. In contrast to some other indicators of successful/unsuccessful ageing (e.g., frailty), these are applicable even in the extreme old. In all age groups, capability, cognition, and mortality were strongly correlated with each other, while multi-morbidity showed no or spurious correlations with any of the other measures of successful ageing (Supplementary Table S1).

We found various associations of individual biomarkers with these descriptors of successful ageing (Supplementary Table S2, Supplementary Figures S3, S4, and data not shown). However, these biomarkers were not independent of each other, in fact, they were highly inter-correlated (Supplementary Table S3). Associations were generally strongest between markers belonging to the same biological or pathological process or domain (Supplementary Table S3). This prompted us to calculate domain scores as combined z-scores for multiple markers within a single domain (inverted and/or log-transformed where necessary, see Methods section) with the aim to describe more comprehensively the relative impact of whole (patho-)biological domains on the ageing process.

Analysing all participants together, most of the examined domains were associated with each other (Supplementary Table S4a). These associations were partly driven by underlying age and were accordingly lost in age-specific analyses (Supplementary Table S4b–d). For instance, the positive association between inflammation and senescence over the whole age range is reversed in centenarians and lost in semi-supercentenarians. On the other hand, haematopoiesis, lipid and glucose metabolism, and liver function generally remained associated with each other throughout the age groups (Supplementary Table S4b–d), suggesting common underlying processes between these domains.

**Fig. 2:**
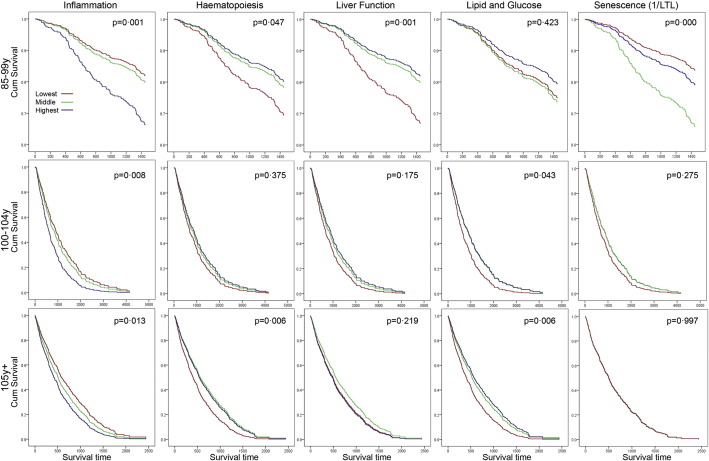
Kaplan–Meier survival curves for tertiles of biomarker domains and telomere length in the very old, centenarian, and (semi-)supercentenarian group. For each age group, the domains were independently organized into tertiles and assessed as independent predictors of survival. The *p*-value indicates the significance of the log-rank Mantel–Cox test of equality of survival distributions.

The associations between biomarker domains and survival in all independent cohorts were tested by Kaplan-Meier analyses of tertiles adjusted for age and gender (Fig. 2). Inflammation was the only domain predicting survival in all age groups, although it did not remain significant in the centenarians (100–104 years) after additional adjustments for education, history of CVD, hypertension, diabetes, hyperlipidaemia, and low albumin (Supplementary Table S5). Cell senescence (telomere length) was associated with survival time in the 85–99 years old group only, but this association was different from expectations as it showed lowest survival for participants in the middle tertile of telomere length. Statin use was not different between the tertiles of telomere length (data not shown). The immunosenescence index could only be calculated in a subset of the (semi-)supercentenarians and did not predict survival in this group (data not shown), although individual immunosenescence markers were associated with survival (Supplementary Figure S4 and Supplementary Table S2c). Renal function was not associated with survival in any of the age groups (not shown).

**Fig. 3:**
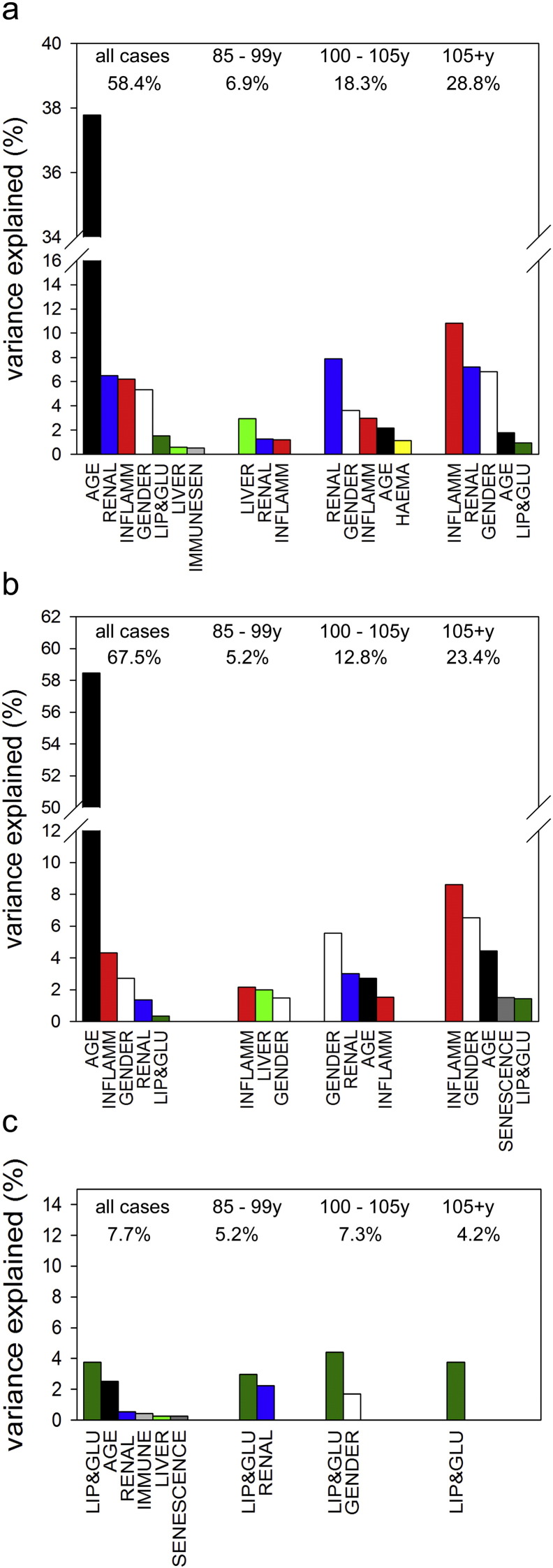
Predictors of capability (a), cognition (b), and multi-morbidity (c). Forward stepwise model selection was performed with age, gender, and all domain scores as predictors. Models were run separately for all unrelated participants and for each of the indicated age groups. Bars indicate the percentage of target variance explained by each predictor. All significant predictors (*p* < 0.05) in each group are shown. The total variance explained by each model is given (as %) on top.

Age group-specific rank correlations showed a persistent and preferential association between the inflammation score and both capability and cognition (but not multimorbidity) over all age groups (Supplementary Table S6). To assess comparatively the impact of different pathophysiological domains on ageing, we performed forward stepwise linear modelling with each of the outcome indicators as target variable and age, gender, and all domain indices as predictor variables. From these models, we calculated the percentage of variation in the outcome measures that was explained by each predictor variable (Figs. 3a–c). In models combining data from all age groups, unsurprisingly age was the most important predictor of capability and cognition. Interestingly, inflammation was the next important predictor of cognition and was only just surpassed in impact for capability by renal function. Combining all age groups, inflammation was a more relevant predictor than gender (Figs. 3a,b). Although renal function predicted capability and cognition in the centenarians (Figs. 3 a,b), the associations were negative (suppl. Tab. S6b, c), probably reflecting the fact that serum creatinine is highly dependent on muscle mass, which is low in the extreme old.

Multi-morbidity alone showed a different pattern. Predictive power of any target variable including age was low, with lipid and glucose metabolism as the prime factor. This remained consistently so when analyses were performed in the age groups separately (Fig. 3c).

Age group-specific analyses showed that despite considerable intra-group age differences, age itself was only exceptionally (i.e. in centenarians) a significant predictor of capability, cognition, or multimorbidity. Importantly, inflammation was the only domain that contributed significantly to the variance of capability and cognition outcomes in all age groups, and its relevance increased with age to become the largest predictor in (semi-)supercentenarians (Figs. 3a–c).

### 3.3 Lower Inflammation Index in Centenarian Offspring

For traditional reasons, there were more males in the offspring group and, associated with male gender, a slightly increased age at enrolment and a higher frequency of smokers (Table 1). The only significant difference in individual biomarkers between offspring and their spouses was a decrease in total cholesterol (Table 1). Although centenarian offspring maintained their telomeres better with age than unrelated participants (Fig. 1) so that their telomere lengths were significantly above the age-dependent mean for unrelated 50 to 99 year olds (Supplementary Figure S1), differences in the senescence indices between centenarian offspring and their spouses did not reach statistical significance (Table 2). This is probably because of the higher abundance of males (with shorter telomeres) in the offspring group. However, a comparison of domain scores revealed that centenarian offspring showed the lowest inflammation index of all groups including their spouses, with the magnitude of the differences increasing with progressing age (Table 2) in line with previous data from a Dutch cohort (Derhovanessian et al., 2010). Centenarian offspring also displayed lower values for the lipid and glucose index than their spouses, but were more similar to the very old and extreme old groups than their spouses for this score (Table 2). There were no significant differences between offspring and spouses in the haematopoiesis, liver and renal function domains.

## 4 Discussion

We aimed to identify biological domains that predict successful ageing at extreme old age and to see whether improved performance in these domains would already be recognizable in centenarian offspring. Thus, we comparatively analysed the association of multiple domains, spanning a range of physiological and pathological processes that may contribute to ageing, with multiple indicators of successful ageing in an unprecedentedly large cohort of centenarians and (semi-)supercentenarians, their offspring and spouses, and an unrelated cohort of the very old. This allowed us to identify low-level inflammation as, after age itself, the most important correlate of not only survival, but also capability and cognition. Over all groups combined and, especially, in the (semi-)supercentenarians, the impact of inflammation on these main indicators of successful ageing is stronger than that of gender, haematopoiesis/anaemia, liver or kidney function, lipid and glucose metabolism, or immune cell senescence. Renal function appeared as a similarly strong predictor of capability, but this was most probably due to its association with low muscle mass. Furthermore, only the inflammation index is consistently lower in centenarian offspring as compared to their spouses and older cohorts (even despite an inverted gender ratio). These results confirm and extend data from younger and/or smaller cohorts (Jenny et al., 2012, Akbaraly et al., 2013, Schnabel et al., 2013, Varadhan et al., 2014). They emphasize the apparent paradox, recognized earlier (Salvioli et al., 2013), that on one hand systemic inflammation is associated with accelerated ageing and enhanced mortality risk, while on the other hand centenarians, who have escaped those risks for longer and age slower than the general population, show high levels of systemic inflammation markers. One approach to explain this was the suggestion that not the total, systemic amount of pro-inflammatory mediators but their tissue- and cell-specific origin and place of action might determine whether they are dangerous for health and longevity or actually good for physiological responses. As another not mutually exclusive possibility, it was suggested that high levels of anti-inflammatory molecules in long-living people may counteract inflamm-ageing, both suggestions implying that high systemic levels of pro-inflammatory molecules would not confer increased risks in centenarians (Salvioli et al., 2013). Our data suggest another interpretation: Seeing low levels of systemic inflammation in centenarian offspring indicated that the rise of inflammatory mediators in centenarians may be a relatively late, ‘catch-up’ event. Thus, future centenarians may be risk-protected over most of their lifespan by low levels of systemic inflammation, but when these levels rise towards the end of their lifespan they predict centenarian mortality, disability and cognitive decline at least as strongly as in the general population.

This interpretation is consistent with results from animal studies. In mice, as in humans, enhanced systemic inflammation is associated with accelerated ageing and increased mortality risk (Osorio et al., 2012, Kawahara et al., 2009, Tilstra et al., 2012, Caballero et al., 2009). Importantly, systemic activation of the major pro-inflammatory transcription factor NF-κB in the absence of any other genetic or environmental factor is sufficient to accelerate ageing in mice, suggesting that chronic enhancement of pro-inflammatory mediators is not just a bystander but a driver of ageing (Jurk et al., 2014).

Together, our results suggest suppression of chronic inflammation as a major determinant of successful longevity, which is relevant over a very wide age range up to extreme old age. Further human studies in independent cohorts of the extreme old will be necessary to address the generalisability of these results.

Anti-inflammatory interventions have proven power to rescue premature ageing in mice (Jurk et al., 2014, Tilstra et al., 2012, Strong et al., 2008). However, serious side effects of available potent anti-inflammatories presently preclude their long-term use in humans (Jaturapatporn et al., 2012, Pellicano, 2014, Rainsford, 2007, Strand, 2007). Potentially, more pleiotropic drugs like melatonin that include anti-inflammatory effects in a wide action spectrum could offer safer alternatives (Caballero et al., 2009, Hardeland, 2013). Our results suggest that the development of more sophisticated and safer anti-inflammatory strategies could be an essential step towards the prevention of human premature ageing.

In our centenarian and (semi-)supercentenarian cohorts, multimorbidity was not associated with other outcome indicators of the ageing process (Barthel index, MMSE or survival time) and was not predicted by markers of inflammation. Significant associations between inflammation markers and multimorbidity in the very old have been found in many (Stepanova et al., 2015) but not all (Martin-Ruiz et al., 2011) previous studies. The operational definition of multimorbidity is a potential problem; previous studies used disease counts including any number from 9 to 35 different items either self-rated, clinician-rated or record-extracted, or a number of more complex scores or scales (Huntley et al., 2012). Due to the problematic nature of clinical diagnoses at extreme old age, we restricted ourselves to a list of only 8 clinically verified diagnoses, omitting for instance arthritis, osteoporosis, pulmonary disease, respiratory disease, thyroid disease, and dementia but including in contrast to many other studies fragility fractures. To test the sensitivity of our conclusions to the specific operational definition of multimorbidity, we calculated alternative disease counts either including diagnosed dementia (given that cognitive status as measured by MMSE is dependent on inflammation score) or excluding frailty fracture (which is the most frequent condition in the over 100's and might be regarded as an accident rather than a chronic disease). However, associations of the so modified disease counts with other ageing outcome indicators or with the inflammation score still remained insignificant in all age groups tested. We conclude that while our multimorbidity measure might be as robust as possible in the extreme old, it still indicates that multimorbidity in our populations is not governed by inflammation and does not co-vary with either survival time, capability or cognitive function. Whether this is because of the extreme age or some other property of our study population can only be assessed by comparison to independent cohorts.

Telomere length in offspring was related to that of their centenarian fathers, but not to that of centenarian mothers. Previous studies demonstrated significant cohort heterogeneity in terms of mother-offspring vs father-offspring telomere length correlations (Eisenberg, 2014), however, it is now well established that paternal but not maternal age at conception predicts offspring telomere length (Broer et al., 2013). It is possible that this paternal age effect explains the father–offspring association seen here.

Cross-sectionally, our data show superior maintenance of telomere length in centenarian offspring, centenarians and (semi-)supercentenarians. They confirm the finding of improved telomere length maintenance in Ashkenazy centenarians (Atzmon et al., 2010) in a genetically independent, large cohort. They raise doubt about the validity of previous claims suggesting equal telomere shortening in centenarians (Kimura et al., 2007) or a fast drop of telomere length between centenarians and semi-supercentenarians (Tedone et al., 2014) that were derived from very low numbers of participants. Together, our data suggest that long telomeres might be a prerequisite for exceptional lifespan in humans. They also suggest that centenarian offspring is able to counteract telomere shortening that occurs in the general population and this might contribute to their higher probability of longevity. It is possible that people with exceptional longevity, who also maintain very low risks of CVD, diabetes, and auto-immune diseases, escape from telomere-driven pathologies by maintaining relatively long telomeres.

Given that inflammation and telomere-dependent cell senescence can drive each other thus causing accelerated ageing (Jurk et al., 2014, Tchkonia et al., 2013), we were surprised to find evidence for inflammation as driver of ageing up to (semi-)supercentenarians, while telomere length/cell senescence was no longer predictive for successful ageing once longevity had been achieved. In a previous study comprising only 38 participants, longer telomeres were associated with better health and independence in centenarians (Terry et al., 2008). However, other studies had shown before that telomere length loses its predictive power as a biomarker of mortality and morbidity risk at ages above 75 years (Cawthon et al., 2003, Martin-Ruiz et al., 2005). Moreover, there were a number of limitations to our assessment of cell senescence. Firstly, there are technical limitations with the reproducibility of telomere length measurements (Martin-Ruiz and von Zglinicki, 2014, Martin-Ruiz et al., 2014). In addition, telomere length is limited as a biomarker of senescence because telomere dysfunction can induce senescence without concomitant telomere shortening (Jurk et al., 2014, Hewitt et al., 2012). Due to biobanking limitations, further senescence markers like p16 could not be included in the study. Second, latent CMV infection is associated with both immunosenescence and chronic inflammation (Sansoni et al., 2014). However, using plasma CMV titers as part of the senescence score did not result in a stronger predictive power of cell senescence for successful ageing (data not shown). We also used T cell surface markers, specifically CD4 and CD28, as an additional indicator of immunosenescence. This approach was unprecedentedly well powered in the semi-supercentenarian group, but we were not able to analyse good numbers of participants from the other study groups. Low proportions of CD28 + naive T cells, indicating higher fractions of memory/senescent T cells in the peripheral blood, predicted decreased survival in the (semi-)supercentenarians. CMV titers were inversely correlated with the proportion of CD28 + cells in the whole study cohort (Supplementary Table 3), but not in (semi-)supercentenarians (r = − 0.110; *p* = 0.193). This might indicate that humans with exceptional longevity are less susceptible to CMV-associated immunosenescence in accordance with earlier studies (Derhovanessian et al., 2010).

There are additional limitations to our study, the most important being that not all domains could be assessed with sufficient participant numbers in all age groups. Moreover, numbers of individual parameters spanning a domain are variable, which might make some domain descriptions less powerful than others. Creatinine-based estimation of glomerular filtration rate (eGFR) by using any formula has not been properly validated in the very old and centenarians due to its strong dependency on muscle mass (Stevens et al., 2006), which limits the predictive power of the kidney function domain. In addition, we are aware of the risk to lose specific information by analysing process domains rather than individual candidate biomarkers. However, we believe that random variation, both technical and biological, is the most important source of differences between biomarker patterns describing the same pathophysiological process. Creating biomarker domains by non-weighted averaging of z scores reduced this random variation.

Despite these limitations, our study showed that over a very wide age range from 45 to 115 years, including unprecedentedly large numbers of the extremely old, inflammation is an important driver of ageing that might be amenable to future pharmacological intervention. Accordingly, designing novel, safe anti-inflammatory or immune-modulating medication has major potential to improve healthy lifespan.

## Funding

The study was supported by a grant from the Ministry of Health, Welfare, and Labour for the Scientific Research Project for Longevity (NH); the Grant-in-Aid for Scientific Research (C) (Nos. 20590706 (MT), 21590775 (YA), 24590898 (YA)) from the Japan Society for the Promotion of Science; the medical-welfare-food-agriculture collaborating consortium project from the Japan Ministry of Agriculture, Forestry, and Fisheries (TT); the Daiwa Anglo-Japanese Foundation (YA); grant G0601333 from the UK Medical Research Council and the Biotechnology and Biological Sciences Research Council (TvZ); a grant from Suntory Global Innovation Centre FY2014 (MS); the NIHR Biomedical Research Centre at Newcastle upon Tyne Hospitals NHS Foundation Trust and Newcastle University (CMR); and the Biobank Japan Program from the Ministry of Education, Culture, Sports, and Technology (NH and MS). YAbe was supported by the medical-welfare-food-agriculture collaborating consortium project from the Japan Ministry of Agriculture, Forestry, and Fisheries.

## Role of the Funding Sources

The sponsors had no role in the conduct or interpretation of the study. YA received research grants from DAIICHI SANKYO Co, Ltd and Takeda Pharmaceutical Company Ltd. NH received a research grant from MSD K.K. SK is a consultant for Medical and Biological Laboratories, Co. Ltd. The authors had no financial relationships with any other organisations that might have an interest in the submitted work in the previous three years, and no other relationships or activities that could appear to have influenced the submitted work.

## Author Contributions

YA, TT, SK, NH, and TvZ conceived the study design. YA, CMR, MT, YAbe, and HN participated in data collection. YA, CMR, MT, YAbe, TT, MS, and TvZ participated in data analysis and interpretation. CMR did the final statistical analysis. YAbe assisted with data preparation. SK provided critical revision of the draft. YA, MS, and TvZ drafted the report. All authors approved the final version of the report.

## Figures and Tables

**Table 1:** Baseline characteristics of the participants.

	Family of centenarians		Very old	Centenarians			
Offspring	Unrelated family	85–99 years	100–104 years	105–109 years	110 +	*p*
N (male, female)	167 (126, 41)	167 (41, 126)	536 (234, 302)	275 (59, 216)	387 (48, 339)	22 (2, 20)	< 0.001^a^
Age at enrollment, median [IQR]	76.5 [71.3, 80.6]	73.2 [68.3, 78.6]	87.4 [86.2, 88.8]	100.7 [100.2, 102.1]	106.8 [105.9, 107.5]	110.7 [110.4, 111.2]	
Living conditions, nursing home N (%)	0 (0.0%)	0 (0.0%)	2 (0.4%)	90 (32.7%)	263 (68.0%)	18 (81.8%)	< 0.001^a^
Past or current smoking, N (%)	87 (52.1%)	33 (19.8%)	205 (38.2%)	39 (14.2%)	37 (9.6%)	2 (9.1%)	< 0.001^a^
Missing data, N (%)	13 (7.8%)	21 (12.6%)	20 (3.7%)	1 (0.4%)	3 (0.8%)	1 (4.5%)	
Higher education, N (%)	NA	NA	195 (36.4%)	58 (21.1%)	40 (10.3%)	2 (9.1%)	< 0.001^a^
Missing data, N (%)	NA	NA	19 (3.5%)	8 (2.9%)	18 (4.7%)	1 (4.5%)	
Barthel index, median [IQR]	100 [100, 100]	100 [100, 100]	100 [95, 100]	45 [15, 80]	15 [5, 50]	5 [0, 15]	
Missing data, N (%)	18 (10.8%)	28 (16.8%)	7 (1.3%)	3 (1.1%)	6 (1.6%)	1 (4.5%)	
MMSE, median [IQR]	NA	NA	27 [25,29]	14 [8,21]	6 [0, 13]	0 [0, 4]	
Missing data, N (%)	NA	NA	7 (1.3%)	34 (12.3%)	92 (23.8%)	4 (18.2%)	
Past medical history, N (%)							
Hypertension	66 (42.0%)	65 (41.4%)	336 (62.7%)	98 (35.9%)	147 (38.4%)	8 (36.4%)	< 0.001^a^
Diabetes mellitus	21 (13.5%)	22 (14.2%)	100 (18.7%)	19 (6.9%)	22 (5.7%)	2 (9.1%)	< 0.001^a^
Hyperlipidaemia	25 (15.0%)	34 (20.4%)	225 (42.0%)	39 (14.2%)	50 (12.9%)	5 (22.7%)	< 0.001^a^
Fragility fracture	9 (5.8%)	12 (7.9%)	123 (23.0%)	126 (46.7%)	212 (55.2%)	11 (50.0%)	< 0.001^a^
Cancer other than skin	20 (12.9%)	10 (6.6%)	100 (18.9%)	29 (10.8%)	45 (11.7%)	4 (18.2%)	0.474^a^
Stroke	15 (9.0%)	6 (3.6%)	92 (17.2%)	45 (16.4%)	75 (19.4%)	2 (9.1%)	< 0.001^a^
Coronary heart disease	11 (6.6%)	6 (3.6%)	54 (10.1%)	40 (14.5%)	58 (15.0%)	3 (13.6%)	< 0.001^a^
Renal disease	6 (3.6%)	9 (5.4%)	57 (10.6%)	22 (8.0%)	27 (7.0%)	0 (0.0%)	< 0.001^a^
No of chronic Conditions, Median [IQR]	1 [0,2]	1 [0,2]	2 [1,3]	1 [1,2]	2 [1,2]	2 [1,2]	
Missing data, N (%)	13 (7.8%)	15 (9.0%)	9 (1.7%)	8 (2.9%)	5 (1.3%)	0 (0.0%)	
Laboratory data, mean ± SD							
WBC, /μL	5860 ± 1569	5610 ± 1293	5752 ± 1344	5386 ± 1425	5459 ± 1730	6301 ± 3331	0.029
RBC, × 106/μL	4.44 ± 0.47	4.33 ± 0.42	4.12 ± 0.44	3.62 ± 0.50	3.61 ± 0.55	3.77 ± 0.61	< 0.001
Haemoglobin, g/dL	13.8 ± 1.4	13.3 ± 1.3	12.5 ± 1.4	11.3 ± 1.5	11.2 ± 1.6	11.6 ± 1.8	< 0.001
Platelets, × 104/μL	21.5 ± 5.5	21.9 ± 5.4	21.7 ± 5.5	18.9 ± 5.8	19.4 ± 6.7	19.3 ± 8.4	< 0.001
Missing data, N (%)	2 (1.2%)	1 (0.6%)	4 (0.7%)	10 (3.6%)	10 (2.6%)	0 (0.0%)	
Total cholesterol, mg/dL	196 ± 35	212 ± 33	201 ± 33	172 ± 34	165 ± 36	156 ± 34	< 0.001
HDL-cholesterol, mg/dL	54 ± 15	58 ± 14	59 ± 15	53 ± 13	45 ± 13	44 ± 13	< 0.001
Albumin, g/dL	4.2 ± 0.3	4.2 ± 0.3	4.1 ± 0.3	3.7 ± 0.4	3.4 ± 0.4	3.2 ± 0.4	< 0.001
Creatinine, mg/dL	0.78 ± 0.17	0.72 ± 0.48	0.84 ± 0.51	0.89 ± 0.45	0.86 ± 0.43	0.78 ± 0.32	0.002
Missing data, N (%)	0 (0.0%)	0 (0.0%)	0 (0.0%)	3 (1.1%)	3 (0.8%)	0 (0.0%)	
CMV positive, N (%)	162 (97.6%)	165 (99.4%)	524 (98.1%)	266 (99.6%)	383 (100.0%)	21 (100.0%)	0.133
CMV titer Median [IQR]	18.1 [11.5, 28.1]	21.6 [14.5, 34.2]	18.8 [12.4, 29.1]	27.7 [17.1, 43.9]	29.6 [18.8, 53.0]	25.2 [16.1, 40.4]	
Missing data, N (%)	1 (0.6%)	1 (0.6%)	2 (0.4%)	8 (2.9%)	4 (1.0%)	1 (4.5%)	
CRP, Median [IQR],mg/dL	0.07 [0.04, 0.14]	0.07 [0.04, 0.16]	0.09 [0.04, 0.19]	0.16 [0.06, 0.44]	0.25 [0.09, 0.80]	0.21 [0.07, 1.15]	< 0.001
Missing data, N (%)	1 (0.6%)	1 (0.6%)	0 (0.0%)	3 (1.1%)	4 (1.0%)	0 (0.0%)	
Interleukin-6, Median [IQR], pg/mL	1.03 [0.65, 1.52]	1.13 [0.78, 1.52]	1.69 [1.29, 2.46]	2.86 [2.26, 4.27]	3.31 [2.41, 5.31]	3.30 [2.40, 6.84]	< 0.001
TNF-alpha, Median [IQR], pg/mL	1.37 [0.86, 2.06]	2.02 [1.39, 2.47]	2.19 [1.88, 2.79]	3.36 [2.82, 4.24]	4.91 [3.85, 6.49]	4.53 [4.00, 5.38]	< 0.001
Missing data, N (%)	3 (1.8%)	1 (0.6%)	3 (0.6%)	13 (5.1%)	13 (3.4%)	1 (4.5%)	
Lymphocyte surface markers							
N (male, female)	17 (15, 2)	17 (2, 15)	NA	8 (0, 8)	111 (11, 100)	3 (0, 3)	
CD4, % Mean ± SD	44.7 ± 10.1	48.7 ± 12.3	NA	39.3 ± 8.9	38.7 ± 11.7	44.0 ± 18.2	
CD8, % Mean ± SD	30.2 ± 10.0	26.4 ± 10.2	NA	30.4 ± 11.0	31.6 ± 11.1	21.6 ± 13.8	
CD4/CD8, median [IQR]	1.37 [1.11, 1.91]	1.88 [1.44, 2.32]	NA	1.02 [0.88, 2.56]	1.20 [0.91, 1.84]	1.62 [0.81, 10.53]	
CD16, % mean ± SD	18.3 ± 9.2	15.3 ± 10.0	NA	33.0 ± 13.6	23.6 ± 14.4	24.4 ± 24.0	
CD28, % mean ± SD	47.8 ± 11.6	48.6 ± 15.2	NA	37.8 ± 9.4	36.9 ± 13.6	48.2 ± 19.9	
CD56, % mean ± SD	24.2 ± 8.2	18.9 ± 10.4	NA	31.5 ± 12.7	28.8 ± 13.8	28.0 ± 30.8	
Telomere length, mean ± SD, bp	3871 ± 621	3773 ± 720	3322 ± 681	3045 ± 761	3387 ± 821	3399 ± 920	
Missing data, N (%)	0 (0.0%)	3 (1.8%)	4 (0.7%)	0 (0.0%)	6 (1.6%)	0 (0.0%)	

a *p*-value is for a two-sided Cochran–Armitage Trend Test.

**Table 2:** Differences in group domain scores to centenarian offspring.

	Offspring against	Mean difference	Std. error	Significance	95% confidence interval	
Lower bound	Upper bound
Haematopoiesis	Unrelated Family	0.573	0.229	0.092	− 0.054	1.199
Very old (85–99 years)	1.238⁎	0.186	0.000	0.730	1.745
Centenarians (100–104 years)	3.146⁎	0.207	0.000	2.581	3.711
(Semi)-Supercentenarians (105 years +)	3.074⁎	0.193	0.000	2.547	3.601
Inflammation	Unrelated Family	− 0.795⁎	0.235	0.006	− 1.436	− 0.154
Very old (85–99 years)	− 1.805⁎	0.190	0.000	− 2.324	− 1.285
Centenarians (100–104 years)	− 4.077⁎	0.213	0.000	− 4.657	− 3.496
(Semi)-Supercentenarians (105 years +)	− 5.278⁎	0.198	0.000	− 5.818	− 4.737
Lipid & Glucose	Unrelated Family	− 0.950⁎	0.230	0.000	− 1.578	− 0.322
Very old (85–99 years)	− 0.384	0.187	0.241	− 0.895	0.127
Centenarians (100–104 years)	1.311⁎	0.208	0.000	0.742	1.879
(Semi)-Supercentenarians (105 years +)	1.732⁎	0.194	0.000	1.202	2.262
Liver function	Unrelated family	0.266	0.264	0.851	− 0.454	0.987
Very old (85–99 years)	0.665⁎	0.214	0.016	0.081	1.249
Centenarians (100–104 years)	2.186⁎	0.237	0.000	1.539	2.833
(Semi)-Supercentenarians (105 years +)	1.932⁎	0.222	0.000	1.327	2.538
Renal function	Unrelated family	− 0.049	0.105	0.991	− 0.336	0.239
Very old (85–99 years)	0.426⁎	0.085	0.000	0.193	0.659
Centenarians (100–104 years)	0.736⁎	0.095	0.000	0.478	0.994
(Semi)-supercentenarians (105 years +)	0.666⁎	0.089	0.000	0.425	0.908
Senescence	Unrelated family	− 0.127	0.104	0.738	− 0.412	0.157
Very old (85–99 years)	− 0.709⁎	0.084	0.000	− 0.938	− 0.480
Centenarians (100–104 years)	− 1.066⁎	0.093	0.000	− 1.320	− 0.813
(Semi)-supercentenarians (105 years +)	− 0.624⁎	0.087	0.000	− 0.862	− 0.386

⁎ p < 0.05.
