# Supplementary material for: Inflammation, But Not Telomere Length, Predicts Successful Ageing at Extreme Old Age: A Longitudinal Study of Semi-supercentenarians
Source: eBioMedicine. 2015 Jul 29;2(10):1549–58. doi: 10.1016/j.ebiom.2015.07.029 (PMC4634197; doi:10.1016/j.ebiom.2015.07.029)
Supplement: Supplementary file 1 — Supplementary material. [file mmc1.pdf]

Supplementary Material

Supplementary Tables S1 – S6

Colour code for all tables:

	p≤0·005, positive correlation
	p≤0·05, positive correlation
	p≤0·005, negative correlation
	p≤0·05, negative correlation

Table S1. Correlations between outcome measures (controlled for gender)

a) ALL CASES		BARTHEL INDEX	DISEASE COUNT	MMSE
DISEASE COUNT	Correlation	0·024		
	Signif (2-t'ld)	0·363		
	df	1493		
MMSE	Correlation	0·840	0·160	
	Signif (2-t'ld)	0·000	0·000	
	df	1092	1086	
SURVIVAL TIME	Correlation	0·498	0·066	0·495
	Signif (2-t'ld)	0·000	0·023	0·000
	df	1217	1210	1090

b) Very Old (85-99y)		BARTHEL INDEX	DISEASE COUNT	MMSE
DISEASE COUNT	Correlation	-0·094		
	Signif (2-t'ld)	0·032		
	df	517		
MMSE	Correlation	0·361	-0·010	
	Signif (2-t'ld)	0·000	0·822	
	df	521	517	
SURVIVAL TIME	Correlation	0·212	0·004	0·150
	Signif (2-t'ld)	0·000	0·926	0·001
	df	522	520	522

c) CENTENARIANS (100-104y)		BARTHEL INDEX	DISEASE COUNT	MMSE
DISEASE COUNT	Correlation	-0·059		
	Signif (2-t'ld)	0·334		
	df	264		
MMSE	Correlation	0·673	0·027	
	Signif (2-t'ld)	0·000	0·676	
	df	238	236	
SURVIVAL TIME	Correlation	0·344	0·042	0·376
	Signif (2-t'ld)	0·000	0·501	0·000
	df	263	258	232

d) (SEMI-) SUPERCENTENARIANS (105y+)		BARTHEL INDEX	DISEASE COUNT	MMSE
DISEASE COUNT	Correlation	0·071		
	Signif (2-t'ld)	0·144		
	df	422		
MMSE	Correlation	0·664	0·079	
	Signif (2-t'ld)	0·000	0·152	
	df	327	327	
SURVIVAL TIME	Correlation	0·287	-0·081	0·299
	Signif (2-t'ld)	0·000	0·095	0·000
	df	422	422	326

Table S2. Age group-specific Cox regressions for individual biomarkers

a) Very Old (85-99y)

	Death from any cause					
	Nº Inividuals	Nº Events	Age & Gender Adjusted		Multivariate[1]	
Telomere length (bp)						
< 2984·4	177	35	1·16 (0·70 - 1·93)		1·07 (0·64 - 1·79)	
2984·4 - 3604·1	177	59	2·20 (1·40 - 3·45)	**	1·97 (1·24 - 3·12)	**
> 3604·1	174	28	1		1	
CMV IgG titer						
< 13·70	179	35	1		1	
13·70 - 25·20	175	38	1·20 (0·76 - 1·91)		1·13 (0·71 - 1·80)	
> 25·20	176	50	1·60 (1·03 - 2·47)	*	1·61 (1·04 - 2·51)	*
TNF-alpha (pg/ml)						
< 1·99	174	28	1		1	
1·99 - 2·52	179	39	1·35 (0·83 - 2·20)		1·22 (0·74 - 2·00)	
> 2·52	176	56	2·10 (1·33 - 3·32)	**	2·01 (1·27 - 3·20)	**
IL-6 (pg/ml)						
< 1·41	176	34	1		1	
1·41 - 2·16	178	43	1·24 (0·79 - 1·95)		1·13 (0·71 - 1·80)	
> 2·16	175	46	1·37 (0·87 - 2·15)		1·23 (0·78 - 1·95)	

Values are expressed as HR (95%CI)

[1] Adjusted for age, gender,education, history of CVD, hypertension, diabetes, hyperlipidemia and low albumine (<3·5gdL)· * p<0·05; ** p<0·005

b). Centenarians (100-104y)

Death from any cause	Nº Inividuals	Nº Events	Age & Gender Adjusted		Multivariate[1]	
**Telomere length (bp)**						
< 2715·2	90	90	0·89 (0·65 - 1·21)		0·85 (0·62 - 1·17)	
2715·2 - 3325·0	88	82	0·93 (0·67 - 1·30)		0·94 (0·67 - 1·31)	
> 3325·0	91	85	1		1	
**CMV IgG titer**						
< 20·43	87	81	1		1	
20·43 - 37·67	88	87	1·15 (0·84 - 1·57)		1·17 (0·85 - 1·62)	
> 37·67	86	83	1·27 (0·93 - 1·74)		1·22 (0·88 - 1·68)	
**TNF-alpha (pg/ml)**						
< 2·98	86	85	1		1	
2·98 - 3·91	86	82	1·16 (0·85 - 1·58)		1·19 (0·87 - 1·63)	
> 3·91	87	83	1·57 (1·12 - 2·19)	*	1·52 (1·08 - 2·14)	*
**IL-6 (pg/ml)**						
< 2·45	86	84	1		1	
2·45 - 3·73	86	82	1·14 (0·84 - 1·55)		1·24 (0·90 - 1·70)	
> 3·73	87	84	1·39 (1·03 - 1·89)	*	1·27 (0·93 - 1·73)	

	c) (Semi-)supercentenarians (105y+)					
	Death from any cause					
	Nº Inividuals	Nº Events	Age & Gender Adjusted		Multivariate[1]	
Telomere length (bp)						
< 2960·8	142	127	0·96 (0·75 - 1·23)		0·97 (0·75 - 1·24)	
2960·9-3697·4	144	131	0·97 (0·76 - 1·24)		0·96 (0·75 - 1·24)	
> 3697·5	142	130	1		1	
CMV IgG titer						
< 22·5	145	130	1		1	
22·5-42·2	142	127	0·98 (0·76 - 1·25)		1·04 (0·81 - 1·35)	
> 42·3	142	131	1·06 (0·82 - 1·36)		1·07 (0·83 - 1·37)	
TNF-alpha (pg/ml)						
< 4·19	140	131	1		1	
4·19-5·56	138	120	1·17 (0·91 - 1·51)		1·17 (0·91 - 1·51)	
> 5·57	139	126	1·46 (1·13 - 1·87)	**	1·38 (1·07 - 1·78)	*
IL-6 (pg/ml)						
< 2·62	139	123	1		1	
2·63-4·42	138	124	1·48 (1·14 - 1·90)	**	1·48 (1·14 - 1·92)	**
> 4·43	140	131	1·82 (1·42 - 2·34)	**	1·69 (1·30 - 2·19)	**
CD4 %						
≤ 36·2	70	69	1·59 (1·13 - 2·24)	**	1·75 (1·23 - 2·50)	**
> 36·2	70	70	1		1	
CD28 %						
< 34·0	70	69	1·49 (1·06 - 2·11)	*	1·84 (1·27 - 2·67)	**
≥ 34·0	70	70	1			

Table S3. Correlations between biomarkers (all cases, controlled for gender)

All Cases		Haematopoiesis/Anaemia				Inflammation				Lipid and Glucose Metabolism					Liver Function			Renal Function		Immunosenescence					
		Hemo globin	RBC	Hae ma to	WBC	CMV titer	IL6	TNFa	CRP	HDL	LDL	Total Chol	Trigl	HbA	AST	ALT	GGTP	Crea tinine	eGFR	CD4	CD8	CD4/8 ratio	CD16	CD28	CD56
RBC count	R	0·897																							
	p	0·000																							
	N	1528																							
Haema tocrit	R	0·976	0·926																						
	p	0·000	0·000																						
	N	1528	1528																						
WBC count	R	0·222	0·259	0·228																					
	p	0·000	0·000	0·000																					
	N	1528	1528	1528																					
CMV titer	R	-0·196	-0·20	-0·19	-0·08																				
	p	0·000	0·000	0·000	0·002																				
	N	1517	1517	1517	1517																				
IL6	R	-0·404	-0·41	-0·40	0·065	0·184																			
	p	0·000	0·000	0·000	0·012	0·000																			
	N	1504	1504	1504	1504	1516																			
TNFa	R	-0·470	-0·48	-0·46	-0·06	0·278	0·606																		
	p	0·000	0·000	0·000	0·015	0·000	0·000																		
	N	1503	1503	1503	1503	1515	1520																		
CRP	R	-0·161	-0·14	-0·16	0·216	0·121	0·533	0·345																	
	p	0·000	0·000	0·000	0·000	0·000	0·000	0·000																	
	N	1523	1523	1523	1523	1531	1518	1517																	
HDLC	R	0·140	0·146	0·140	-0·02	-0·16	-0·30	-0·36	-0·28																
	p	0·000	0·000	0·000	0·342	0·000	0·000	0·000	0·000																
	N	1527	1527	1527	1527	1533	1520	1519	1545																
LDLC	R	0·295	0·313	0·302	0·066	-0·11	-0·32	-0·24	-0·18	0·182															
	p	0·000	0·000	0·000	0·010	0·000	0·000	0·000	0·000	0·000															
	N	1517	1517	1517	1517	1523	1510	1509	1535	1539															
Total Chole sterol	R	0·387	0·406	0·393	0·117	-0·17	-0·44	-0·39	-0·26	0·434	0·899														
	p	0·000	0·000	0·000	0·000	0·000	0·000	0·000	0·000	0·000	0·000														
	N	1527	1527	1527	1527	1533	1520	1519	1545	1549	1539														
Triglyc	R	0·337	0·349	0·340	0·233	-0·06	-0·24	-0·23	-0·06	-0·21	0·256	0·400													
	p	0·000	0·000	0·000	0·000	0·012	0·000	0·000	0·020	0·000	0·000	0·000													
	N	1527	1527	1527	1527	1533	1520	1519	1545	1549	1539	1549													
HbA1c	R	0·163	0·254	0·188	0·168	-0·12	-0·15	-0·22	-0·05	0·066	0·131	0·210	0·265												

	p	0·000	0·000	0·000	0·000	0·000	0·000	0·000	0·041	0·011	0·000	0·000	0·000												
	N	1513	1513	1513	1513	1512	1499	1498	1513	1515	1505	1515	1515												
AST	R	0·179	0·133	0·160	0·002	0·003	-0·08	-0·03	-0·06	0·154	0·020	0·091	0·056	0·057											
	p	0·000	0·000	0·000	0·951	0·917	0·003	0·188	0·014	0·000	0·429	0·000	0·028	0·026											
	N	1527	1527	1527	1527	1533	1520	1519	1545	1549	1539	1549	1549	1515											
ALT	R	0·413	0·388	0·398	0·123	-0·15	-0·33	-0·34	-0·15	0·176	0·147	0·259	0·230	0·271	0·661										
	p	0·000	0·000	0·000	0·000	0·000	0·000	0·000	0·000	0·000	0·000	0·000	0·000	0·000	0·000										
	N	1527	1527	1527	1527	1533	1520	1519	1545	1549	1539	1549	1549	1515	1549										
GGTP	R	0·370	0·315	0·352	0·184	-0·12	-0·20	-0·27	0·082	0·109	0·131	0·220	0·236	0·202	0·345	0·533									
	p	0·000	0·000	0·000	0·000	0·000	0·000	0·000	0·001	0·000	0·000	0·000	0·000	0·000	0·000	0·000									
	N	1527	1527	1527	1527	1533	1520	1519	1545	1549	1539	1549	1549	1515	1549	1549									
Crea tinine	R	-0·120	-0·14	-0·12	0·066	0·011	0·095	0·204	0·097	-0·14	-0·05	-0·08	0·066	0·054	-0·019	-0·04	0·081								
	p	0·000	0·000	0·000	0·010	0·654	0·000	0·000	0·000	0·000	0·059	0·003	0·009	0·035	0·461	0·122	0·002								
	N	1527	1527	1527	1527	1533	1520	1519	1545	1549	1539	1549	1549	1515	1549	1549	1549								
eGFR	R	0·327	0·330	0·314	0·003	-0·14	-0·23	-0·38	-0·14	0·157	0·039	0·096	0·000	0·017	0·068	0·198	0·094	-0·87							
	p	0·000	0·000	0·000	0·892	0·000	0·000	0·000	0·000	0·000	0·122	0·000	0·998	0·511	0·008	0·000	0·000	0·000							
	N	1527	1527	1527	1527	1533	1520	1519	1545	1549	1539	1549	1549	1515	1549	1549	1549	1549							
CD4	R	0·179	0·167	0·174	-0·05	-0·14	-0·15	-0·21	-0·20	-0·01	0·123	0·133	0·143	0·005	0·026	0·092	0·088	-0·15	0·201						
	p	0·025	0·037	0·030	0·552	0·088	0·066	0·010	0·011	0·938	0·128	0·100	0·076	0·948	0·750	0·257	0·275	0·055	0·012						
	N	156	156	156	156	156	155	155	155	155	155	155	155	152	155	155	155	155	155						
CD8	R	-0·184	-0·18	-0·19	-0·05	0·070	0·034	0·150	0·001	-0·05	-0·06	-0·08	0·019	-0·01	-0·077	-0·07	-0·15	0·111	-0·16	-0·43					
	p	0·022	0·021	0·016	0·571	0·388	0·674	0·062	0·991	0·578	0·442	0·310	0·810	0·904	0·340	0·418	0·056	0·170	0·051	0·000					
	N	156	156	156	156	156	155	155	155	155	155	155	155	152	155	155	155	155	155	156					
CD4/CD 8 ratio	R	0·215	0·197	0·214	-0·01	-0·13	-0·10	-0·20	-0·09	0·016	0·088	0·108	0·046	0·006	0·069	0·084	0·143	-0·21	0·257	0·797	-0·86				
	p	0·007	0·014	0·007	0·908	0·100	0·217	0·012	0·261	0·845	0·276	0·180	0·571	0·944	0·394	0·299	0·076	0·011	0·001	0·000	0·000				
	N	156	156	156	156	156	155	155	155	155	155	155	155	152	155	155	155	155	155	156	156				
CD16	R	-0·108	-0·10	-0·12	-0·04	0·005	0·161	0·156	0·114	0·065	-0·13	-0·11	-0·19	0·004	0·019	-0·09	-0·16	0·049	-0·09	-0·67	0·183	-0·47			
	p	0·180	0·204	0·147	0·598	0·946	0·045	0·052	0·156	0·424	0·112	0·186	0·018	0·961	0·810	0·272	0·042	0·544	0·277	0·000	0·022	0·000			
	N	156	156	156	156	156	155	155	155	155	155	155	155	152	155	155	155	155	155	156	156	156			
CD28	R	0·232	0·191	0·224	-0·08	-0·20	-0·24	-0·31	-0·31	0·019	0·115	0·129	0·136	0·005	-0·024	0·081	0·138	-0·23	0·297	0·809	-0·45	0·715	-0·55		
	p	0·004	0·017	0·005	0·342	0·015	0·002	0·000	0·000	0·816	0·154	0·109	0·092	0·953	0·771	0·318	0·087	0·004	0·000	0·000	0·000	0·000	0·000		
	N	156	156	156	156	156	155	155	155	155	155	155	155	152	155	155	155	155	155	156	156	156	156		
CD56	R	-0·126	-0·13	-0·14	-0·03	0·090	0·173	0·153	0·172	0·063	-0·13	-0·11	-0·10	-0·09	0·062	0·019	-0·08	0·039	-0·05	-0·68	0·223	-0·49	0·694	-0·59	
	p	0·116	0·116	0·092	0·685	0·266	0·032	0·057	0·032	0·433	0·109	0·167	0·198	0·299	0·441	0·819	0·320	0·631	0·541	0·000	0·005	0·000	0·000	0·000	
	N	156	156	156	156	156	155	155	155	155	155	155	155	152	155	155	155	155	155	156	156	156	156	156	
Telo Length	R	0·176	0·180	0·173	-0·01	-0·05	-0·17	-0·15	-0·07	0·014	0·090	0·100	0·093	0·036	0·042	0·129	0·060	-0·08	0·112	0·246	-0·17	0·237	-0·24	0·270	-0·22
	p	0·000	0·000	0·000	0·778	0·054	0·000	0·000	0·007	0·571	0·000	0·000	0·000	0·165	0·103	0·000	0·017	0·003	0·000	0·002	0·039	0·003	0·003	0·001	0·005
	N	1515	1515	1515	1515	1524	1508	1507	1532	1536	1526	1536	1536	1503	1536	1536	1536	1536	1536	155	155	155	155	155	155

Tab S4. Correlations between domain indices (controlled for gender)

a) All Cases		Haematopoiesis	Inflammation	Lipid and Glucose	Liver Function	Renal Function
Inflammation	R	-0·265				
	p	0·000				
	df	1516				
Lipid and Glucose	R	0·397	-0·377			
	p	0·000	0·000			
	df	1526	1501			
Liver Function	R	0·295	-0·128	0·196		
	p	0·000	0·000	0·000		
	df	1551	1530	1529		
Renal Function	R	0·243	-0·215	0·037	0·098	
	p	0·000	0·000	0·154	0·000	
	df	1524	1504	1502	1546	
Senescence	R	-0·135	0·134	-0·092	-0·069	-0·111
	p	0·000	0·000	0·000	0·006	0·000
	df	1539	1517	1516	1560	1533

b) Very Old (85-99y)		Haematopoiesis	Inflammation	Lipid and Glucose	Liver Function	Renal Function
Inflammation	R	0·024				
	p	0·577				
	df	528				
Lipid and Glucose	R	0·217	0·013			
	p	0·000	0·758			
	df	524	524			
Liver Function	R	0·122	0·006	0·113		
	p	0·005	0·896	0·009		
	df	529	530	525		
Renal Function	R	0·065	-0·151	-0·066	0·000	
	p	0·136	0·000	0·132	0·996	
	df	529	530	525	533	
Senescence	R	0·022	0·091	0·027	0·013	0·004
	p	0·615	0·036	0·540	0·773	0·923
	df	525	526	521	529	529

c) Centenarians (100-104y)		Haematopoiesis	Inflammation	Lipid and Glucose	Liver Function	Renal Function
Inflammation	R	-0·025				
	p	0·698				
	df	247				
Lipid and Glucose	R	0·339	-0·184			
	p	0·000	0·003			
	df	256	248			
Liver Function	R	0·223	0·021	0·054		
	p	0·000	0·736	0·386		
	df	262	254	257		
Renal Function	R	0·259	-0·125	-0·016	0·007	
	p	0·000	0·046	0·802	0·911	
	df	262	254	257	270	
Senescence	R	-0·001	-0·147	0·050	0·008	0·053
	p	0·993	0·019	0·423	0·899	0·385
	df	262	254	257	270	270

d) (Semi-) Supercentenarians (105y+)		Haematopoiesis	Inflammation	Lipid and Glucose	Liver Function	Renal Function	Senescence
Inflammation	R	0·068					
	p	0·168					
	df	410					
Lipid and Glucose	R	0·313	-0·273				
	p	0·000	0·000				
	df	418	407				
Liver Function	R	0·225	0·077	0·099			
	p	0·000	0·117	0·042			
	df	423	414	419			
Renal Function	R	0·118	0·049	-0·162	0·010		
	p	0·018	0·338	0·001	0·846		
	df	396	388	392	403		
Senescence	R	-0·107	-0·044	-0·024	-0·030	-0·101	
	p	0·028	0·375	0·632	0·543	0·043	
	df	418	408	413	424	397	
Immunosenescence	R	-0·030	-0·024	0·041	-0·001	-0·125	0·500
	p	0·727	0·784	0·631	0·992	0·191	0·000
	df	138	134	135	137	110	138

Table S5. Age group-specific Cox regressions for biomarker domains

a) Very Old (85-99y)

Death from any cause	Nº Inividuals	Nº Events	Age & Gender Adjusted		Multivariate[1]	
Haematopoiesis						
< (-0·19)	172	49	1·67 (1·06 - 2·64)	*	1·47 (0·92 - 2·37)	
(-0·19) - (1·38)	180	39	1·13 (0·71 - 1·79)		1·02 (0·64 - 1·63)	
> (1·38)	176	34	1		1	
Inflammation						
< (-1·96)	177	31	1		1	
(-1·96) - (-0·48)	174	34	1·10 (0·68 - 1·79)		1·11 (0·68 - 1·81)	
> (-0·48)	178	58	1·95 (1·25 - 3·03)	**	1·89 (1·21 - 2·95)	**
Lipid & Glucose						
< (-0·12)	174	42	1·12 (0·71 - 1·77)		0·87 (0·52 - 1·47)	
(-0·12) - (1·49)	175	45	1·27 (0·82 - 1·98)		1·02 (0·62 - 1·68)	
> (1·49)	175	35	1		1	
Liver Function						
< (-0·55)	177	58	1·95 (1·25 - 3·04)	**	1·89 (1·21 - 2·97)	**
(-0·55) - (1·02)	177	34	1·10 (0·67 - 1·78)		1·04 (0·64 - 1·71)	
> (1·02)	178	31	1		1	
Renal Function						
< (-0·28)	178	41	0·97 (0·63 - 1·50)		1·05 (0·67 - 1·63)	
(-0·28) - (0·34)	177	41	0·99 (0·64 - 1·53)		0·99 (0·64 - 1·53)	
> (0·34)	177	41	1		1	
Senescence						
< (-0·27)	174	28	1		1	
(-0·27) - (0·53)	177	59	2·20 (1·40 - 3·45)	**	1·97 (1·24 - 3·13)	**
> (0·53)	177	35	1·16 (0·70 - 1·93)		1·07 (0·64 - 1·79)	

Values are expressed as HR (95%CI)

[1] Adjusted for age, gender,education, history of CVD, hypertension, diabetes, hyperlipidemia, and low albumine (<3·5gdL) * p<0·05; ** p<0·005

b)
Centenarians
(100-104y)

Death from any cause

	Nº Inividuals	Nº Events	Age & Gender Adjusted		Multivariate[1]	
**Haematopoiesis**						
< (-2·09)	87	85	1·28 (0·93 - 1·76)		1·02 (0·72 - 1·44)	
(-2·09) - (-0·42)	89	85	1·07 (0·78 - 1·46)		0·95 (0·68 - 1·31)	
> (-0·42)	83	77	1		1	
**Inflammation**						
< 0·05	84	81	1		1	
0·05 - 2·04	85	82	1·13 (0·83 - 1·54)		1·02 (0·73 - 1·40)	
> 2·04	85	82	1·56 (1·14 - 2·14)	**	1·27 (0·91 - 1·78)	
**Lipid & Glucose**						
< (-1·76)	84	80	1·31 (0·95 - 1·82)		0·90 (0·62 - 1·33)	
(-1·76) - (-0·22)	88	87	0·96 (0·70 - 1·31)		0·83 (0·59 - 1·19)	
> (-0·22)	82	77	1		1	
**Liver Function**						
< (-1·89)	89	85	1·50 (1·09 - 2·07)	*	1·32 (0·95 - 1·83)	
(-1·89) - (-0·35)	90	87	1·24 (0·90 - 1·70)		1·24 (0·90 - 1·70)	
> (-0·35)	88	83	1		1	
**Renal Function**						
< (-0·82)	91	85	1·20 (0·88 - 1·64)		1·38 (1·00 - 1·91)	*
(-0·82) - (0·14)	88	87	1·04 (0·76 - 1·42)		1·08 (0·79 - 1·48)	
> (0·14)	88	83	1		1	
**Senescence**						
< 0·09	91	85	1		1	
0·09 - 0·88	88	82	0·93 (0·67 - 1·30)		0·94 (0·67 - 1·31)	
> 0·88	90	90	0·89 (0·65 - 1·21)		0·85 (0·62 - 1·17)	

	c) (Semi-)supercentenarians (105y+)					
	Death from any cause					
	Nº Inividuals	Nº Events	Age & Gender Adjusted		Multivariate[1]	
Haematopoiesis						
< (-2·25)	143	132	1·40 (1·09 - 1·80)	*	1·33 (1·03 - 1·71)	*
(-2·25) - (-0·31)	141	125	0·95 (0·74 - 1·21)		0·98 (0·76 - 1·27)	
>(-0·306)	141	127	1		1	
Inflammation						
< 1·25	139	122	1		1	
1·25 - 3·24	138	125	1·19 (0·93 - 1·54)		1·17 (0·90 - 1·52)	
> 3·24	139	129	1·45 (1·13 - 1·87)	**	1·36 (1·05 - 1·78)	*
Lipid & Glucose						
< (-2·25	140	131	1·46 (1·14 - 1·88)	**	1·35 (1·03 - 1·77)	*
(-2·25) - (-0·51)	139	124	1·10 (0·86 - 1·41)		1·06 (0·82 - 1·38)	
> -0·51	141	124	1		1	
Liver Function						
< (-2·11)	144	133	0·98 (0·77 - 1·26)		0·90 (0·70 - 1·15)	
(-2·11) - (-0·29)	144	129	0·83 (0·65 - 1·06)		0·81 (0·63 - 1·04)	
> (-0·29)	143	128	1		1	
Renal Function						
<-0·84	134	118	0·87 (0·67 - 1·12)		0·92 (0·71 - 1·20)	
(-0·84) - (0·03)	135	120	0·76 (0·59 - 0·98)	*	0·81 (0·63 - 1·05)	
>0·03	135	126	1		1	
Senescence						
<(-0·39)	142	130	1		1	
(-0·39) - (0·56)	144	131	0·98 (0·77 - 1·25)		0·97 (0·75 - 1·24)	
> 0·56	142	127	0·96 (0·75 - 1·24)		0·97 (0·75 - 1·25)	
ImmunoSenescence						
< 0·50	70	70	1		1	
≥ 0·50	70	69	1·19 (0·84 - 1·69)		1·39 (0·96 - 2·02)	

Table S6. Spearman's rank correlations between biological domains and outcome indicators (controlled for gender)

a) Very Old (85-99y)		Haematopoiesis	Inflammation	Lipid & Gluc	Liver Function	Renal	Senescence
BARTHEL INDEX	Correlation	0·106	-0·138	0·089	0·157	-0·054	-0·062
	Signif (2-t'ld)	0·015	0·001	0·043	0·000	0·217	0·157
	df	522	523	518	526	526	522
DISEASE COUNT	Correlation	0·029	0·016	0·160	0·037	-0·129	0·034
	Signif (2-t'ld)	0·512	0·719	0·000	0·394	0·003	0·439
	df	520	521	516	524	524	520
MMSE	Correlation	0·070	-0·151	0·038	0·160	0·002	-0·040
	Signif (2-t'ld)	0·108	0·001	0·388	0·000	0·969	0·364
	df	522	523	518	526	526	522

b) Centenarians (100-104y)		Haematopoiesis	Inflammation	Lipid & Gluc	Liver Function	Renal	Senescence
BARTHEL INDEX	Correlation	0·035	-0·204	0·028	0·065	-0·274	-0·042
	Signif (2-t'ld)	0·575	0·001	0·652	0·288	0·000	0·489
	df	259	251	254	267	267	269
DISEASE COUNT	Correlation	0·151	-0·029	0·267	-0·012	-0·047	0·014
	Signif (2-t'ld)	0·016	0·650	0·000	0·847	0·446	0·822
	df	254	248	251	262	262	264
MMSE	Correlation	-0·013	-0·142	0·038	0·054	-0·155	0·076
	Signif (2-t'ld)	0·841	0·031	0·569	0·406	0·016	0·241
	df	230	229	230	237	237	238

c) (Semi-) Supercentenarians (105y+)		Haematopoiesis	Inflammation	Lipid & Gluc	Liver Function	Renal	Senescence	Immuno-sen
BARTHEL INDEX	Correlation	-0·007	-0·374	0·224	-0·007	-0·284	-0·029	0·048
	Signif (2-t'ld)	0·892	0·000	0·000	0·880	0·000	0·555	0·578
	df	417	409	412	423	396	420	137
DISEASE COUNT	Correlation	0·060	-0·004	0·217	0·097	-0·071	0·018	-0·024
	Signif (2-t'ld)	0·224	0·942	0·000	0·046	0·155	0·716	0·784
	df	417	408	412	423	398	420	135
MMSE	Correlation	-0·051	-0·359	0·223	-0·055	-0·149	-0·090	0·066
	Signif (2-t'ld)	0·361	0·000	0·000	0·317	0·008	0·101	0·523
	df	323	317	321	328	308	327	95

Supplementary Figures

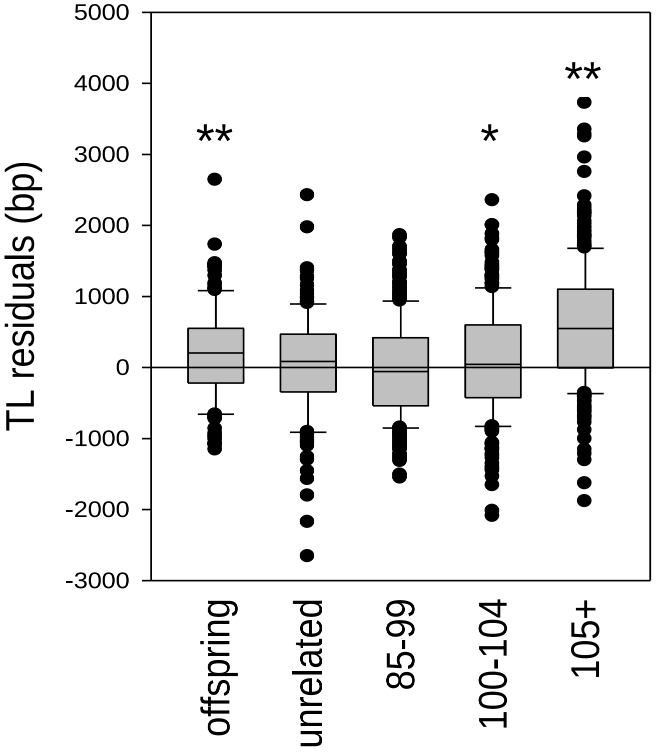

Supplementary Figure S1. Longer telomeres in centenarians, (semi-)supercentenarians, and centenarian offspring. Group-specific telomere length residuals were calculated as differences to length as predicted by the regression line for unrelated individuals aged up to 100 years. Box plots show medians, upper and lower quartiles (boxes), upper and lower centiles (whiskers), and outliers (dots) for each age group. * p < 0·05, ** p < 0·001 for difference to zero.

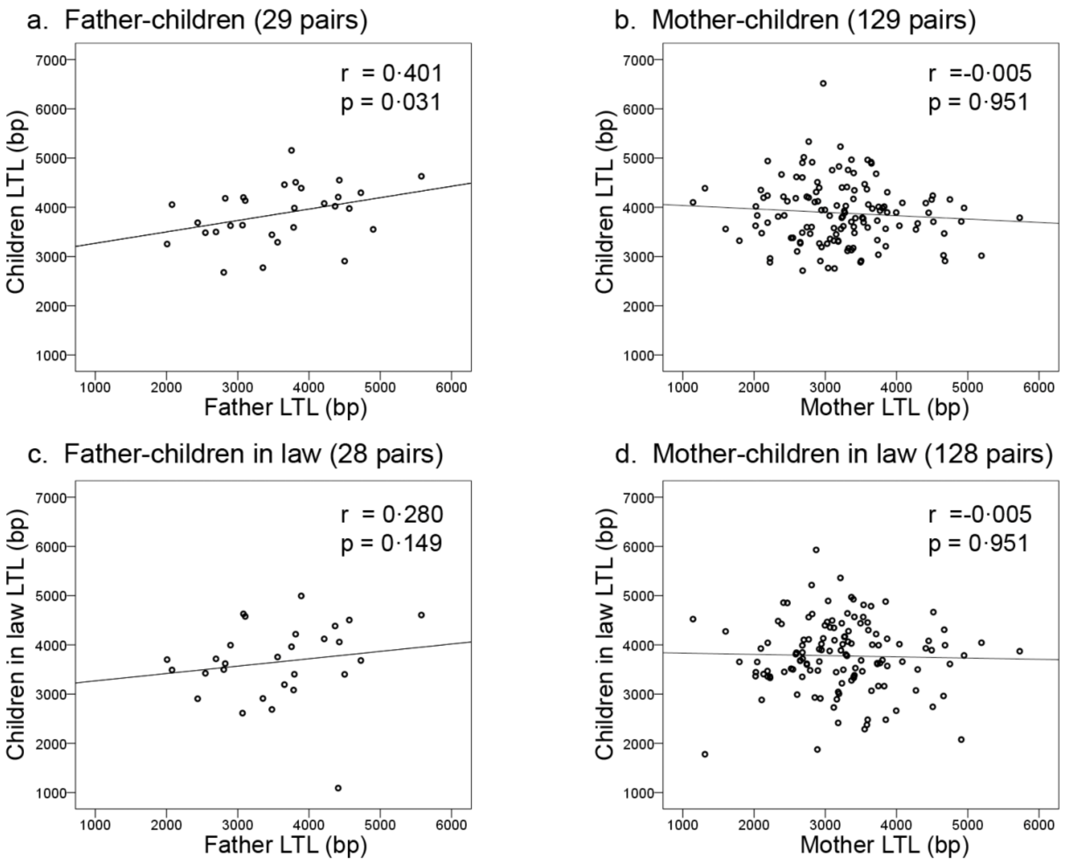

Supplementary Figure S2. Analysis of inter-generational correlations in telomere length. Inter-generational correlations in leukocyte telomere length between centenarian fathers and their children (a), between mothers and their children (b), and between fathers (c) or mothers (d) and their children in law. Spearman's rank correlation coefficients r and error probabilities p are given. The correlations between male centenarians and their offspring remained significant after adjustment for participant age (r = 0·387, p = 0·038).

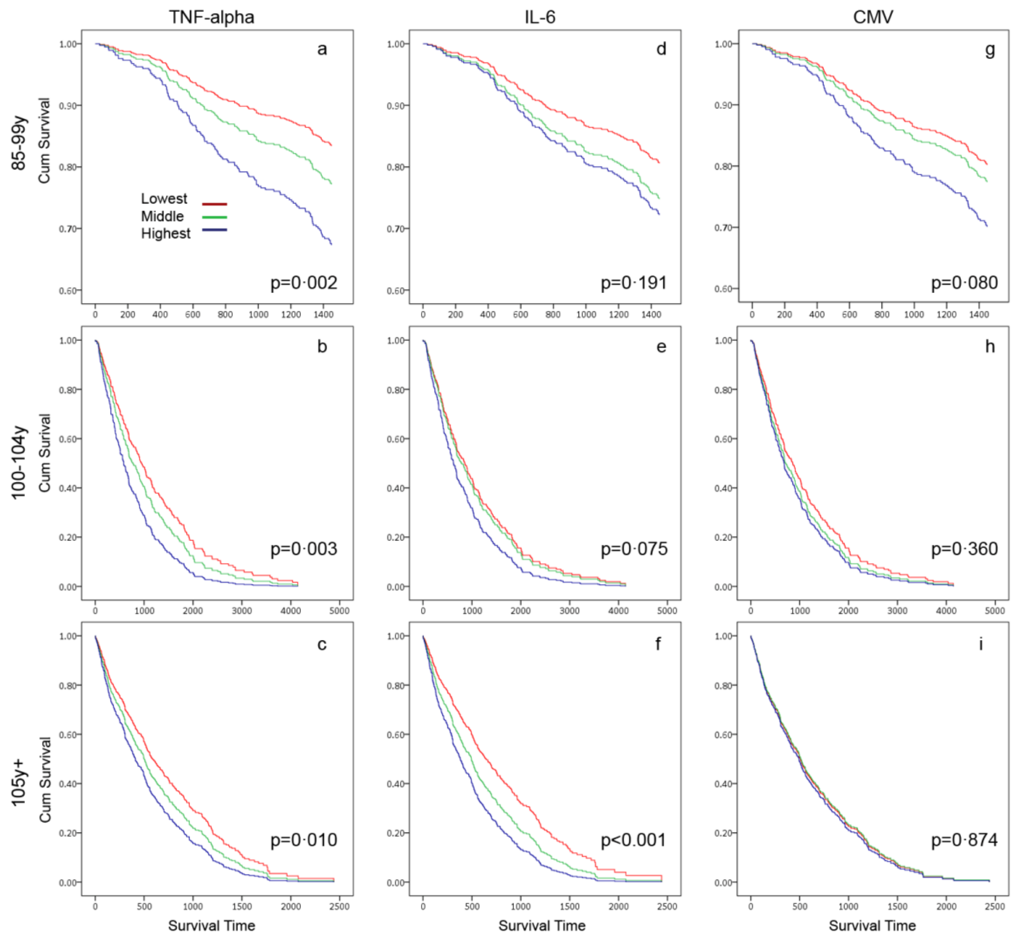

Supplementary Figure S3. Kaplan-Meier survival curves for biomarker tertiles in the very old, centenarian, and (semi-)supercentenarian groups. For each age group, the biomarkers were independently organized into tertiles and assessed as independent predictors of survival. The p-value indicates the significance of the log-rank Mantel-Cox test of equality of survival distributions. Survival time is given in days.

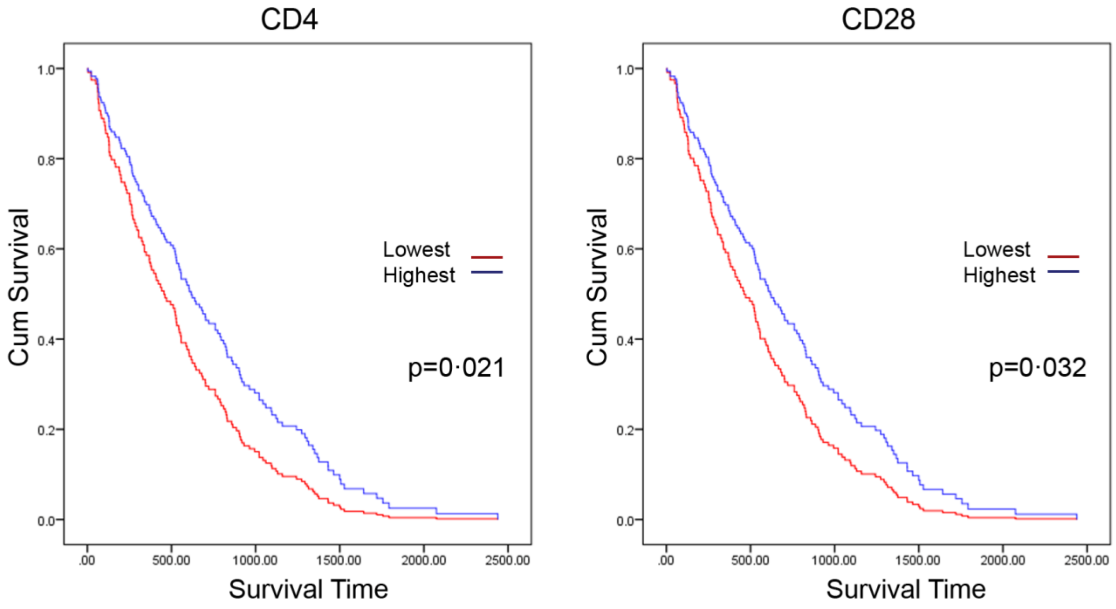

Supplementary Figure S4. Kaplan-Meier survival curves for CD4+ and CD28+ cell frequencies in the (semi-)supercentenarian group. The immunosenescence markers CD4+ and CD28+ (cell frequencies in %) were dichotomized by median because of low data numbers. The p-value indicates the significance of the log-rank Mantel-Cox test of equality of survival distributions. Survival time is given in days.
